# Supplementary material for: Teaching Diagnostic Reasoning to Faculty Using an Assessment for Learning Tool: Training the Trainer
Source: MedEdPORTAL. 2020 Aug 21;16:10938. doi: 10.15766/mep_2374-8265.10938 (PMC7449581; doi:10.15766/mep_2374-8265.10938)
Supplement: Supplementary file 1 — Workshop Agenda.docxFacilitator Guide.docxProblem Representation Exercise.docxCognitive Bias Exercise.docxDiagnostic Reasoning Role-Play.docxPowerPoint Presentation for 2-Hour Workshop.pptxAssessment of Reasoning Tool.pdfLive Example - Poor Diagnostic Reasoning.movLive Example - Excellent Diagnostic Reasoning.movPowerPoint Presentation for 1-Hour Workshop.pptxIdeal Evaluation Form.docx [file mep_2374-8265.10938-s001.zip › _Educational_Summary_Report_10938.pdf]

MedEdPORTAL® The AAMC Journal of Teaching and Learning Resources

Original Publication Open Access

Teaching Diagnostic Reasoning to Faculty Using an Assessment for Learning Tool: Training the Trainer

Adam Cohen, MD*, Moushumi Sur, MD, Martin Weisse, MD, Kathryn Moffett, MD, Jeffrey Lancaster, MD, Renee Saggio, MD, Geeta Singhal, MD, MEd, Satid Thammasitboon, MD, MHPE

*Corresponding author: adcohen.md@gmail.com

Abstract

Introduction: There is a need for a standardized approach to understand and assess clinical reasoning in medical learners. The Assessment of Reasoning Tool was developed based on prevalent theories and frameworks using a multidisciplinary expert panel. As the tool provides a standardized rubric for assessing clinical reasoning, we designed an interactive train-the-trainer workshop for clinical educators and education leaders interested in improving their teaching skills and/or introducing curricula surrounding diagnostic reasoning. **Methods:** In this workshop, participants were exposed to the major domains of diagnostic reasoning and how to apply it to the assessment of a learner's skills. Kolb's experiential learning was the underlying model, which we showcased by using multiple interactive techniques, including small-group discussion, peer sharing, and case practice. We presented the workshop at a national conference of pediatric educators and as a faculty development workshop at a single institution. Participants were asked to complete a survey after the workshop to gauge their reactions and look for areas of improvement. **Results:** A total of 34 participants attended the two workshops. Participants rated the workshop favorably, with most planning to make a change to their practice. Comments were largely positive, emphasizing the benefits of the interactive approach. **Discussion:** The workshop and teaching materials represent an important early step in the workplace-based assessment of diagnostic reasoning in medical learners. Grounded in the clinical reasoning literature, the workshop offers one approach to assessing these skills in learners with or without direct observation of clinical skills.

Keywords
Learner Assessment, Feedback, Clinical Reasoning, Diagnostic Reasoning, Faculty Development, Competency-Based Medical Education, Competencies, Milestones, EPAs, Continuing Professional Development

Educational Objectives

By the end of this activity, learners will be able to:

1. Describe the cognitive processes that influence diagnostic reasoning.
2. Define the five domains of diagnostic reasoning and their related components.
3. Apply a theory-informed instrument to enhance the assessment and feedback of diagnostic reasoning in a medical learner.

Citation:
Cohen A, Sur M, Weisse M, et al. Teaching diagnostic reasoning to faculty using an assessment for learning tool: training the trainer. *MedEdPORTAL.* 2020;16:10938.
https://doi.org/10.15766/mep_2374-8265.10938

Introduction

The Institute of Medicine has published a landmark report on diagnostic errors in health care highlighting the persistence of diagnostic errors and specifically recommending that training programs address diagnostic reasoning performance.[1] While accurate diagnoses are linked to positive patient outcomes, the diagnostic reasoning process is inherently complex and uniquely experiential for each clinician.[2] In addition, the problem-solving aspects are poorly understood, and expert reasoning is not adequately taught. However, using a standardized approach to teaching and assessing the diagnostic reasoning process can enable learners to develop these essential skills for lifelong learning towards diagnostic expertise.

We designed this workshop to develop faculty skills in teaching diagnostic reasoning. Instead of teaching theoretical concepts and their associated skills, we use a theory-informed assessment tool (the Assessment of Reasoning Tool [ART]) as a framework

Copyright © 2020 Cohen et al. This is an open-access publication distributed under the terms of the Creative Commons Attribution license.

for the workshop. The ART is an assessment for learning tool created by a multispecialty team of experts in the Society to Improve Diagnosis in Medicine. The society developed the ART based on prevalent clinical reasoning–related theories and frameworks, contemporary practice goals, and previously proposed error reduction strategies.[3] As such, the ART is a tool that provides a standardized structure for teaching and assessing diagnostic reasoning skills. Clinical teachers can use the ART to provide explicit feedback to their learners and use the framework to create a teaching moment pertaining to the diagnostic reasoning process. The tool is specifically designed to be used during an oral case presentation and therefore does not require direct observation of clinical skills to be utilized.

Although there have been many assessment tools developed for clinical reasoning,[3,4] the ART is unique in that it is specific to diagnostic reasoning, provides the appropriate shared language and theoretical framework to allow for accurate assessment, and is the first tool to specifically evaluate for metacognition. While videos are available online to help faculty develop an understanding of the domains and terminology used in the tool,[5] no currently published work exists that can serve as a guide to faculty development in the use of this tool. Furthermore, while there are some publications in *MedEdPORTAL* regarding feedback associated with diagnostic reasoning,[6-8] none are applicable to both the general medical learner and the presentation of a new case without direct observation of clinical skills.

We created this workshop as an interactive 2-hour faculty development train-the-trainer session for clinical teachers to improve their teaching skills as well as for medical education leaders planning to develop a clinical reasoning curriculum. We designed the workshop around Kolb's experiential learning model[9,10] to create an interactive and practical experience (Figure).

Methods

We initially conducted the 2-hour workshop at the 2019 Council on Medical Student Education in Pediatrics (COMSEP) annual meeting after being selected through a peer-review process. We subsequently modified it to a 1-hour workshop and presented it at a large teaching hospital in the context of a faculty

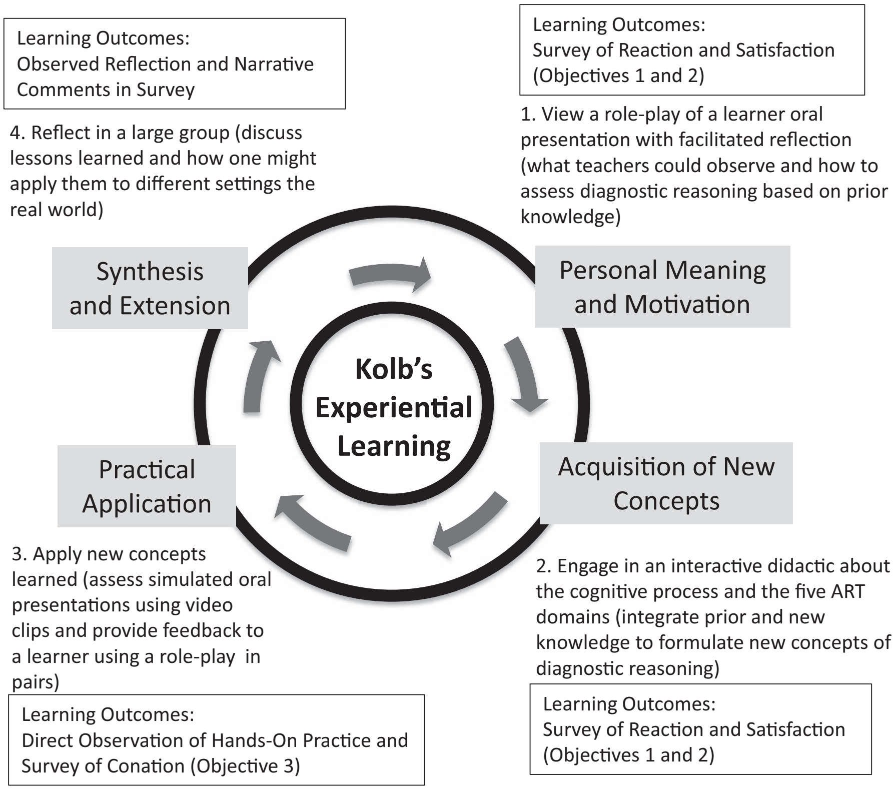

Figure. Kolb's experiential learning cycle as a conceptual framework for the workshop design and the alignments of learning objectives, workshop activities, and learning outcomes. ART, Assessment of Reasoning Tool.

Copyright © 2020 Cohen et al. This is an open-access publication distributed under the terms of the Creative Commons Attribution license.

development session. No previous knowledge or experience was required in order to attend the sessions.

Workshop facilitators were faculty and fellows at major academic medical institutions with experience in teaching the concepts of clinical reasoning. Facilitators were familiar with the conceptual framework of the diagnostic reasoning process provided by Thammasitboon et al.,[3] which included five domains: hypothesis-driven data gathering, problem representation, prioritized differential diagnosis, directing evaluation and treatment towards high-priority diagnoses, and metacognition. As all workshop facilitators were involved in the workshop design, no additional training was needed. The initial target audience at the 2019 COMSEP annual meeting was medical educators interested in teaching and assessing skills in clinical reasoning as well as education leaders looking to improve the quality of clinical reasoning feedback given to learners within their training program. When the workshop was modified specifically for an institutional faculty development exercise, the intended audience was any faculty member with a role in precepting and assessing clinical learners. Of note, while both workshops were presented to pediatric educators, the content was not specialty specific. In fact, the workshop was designed to improve the teaching ability of all clinical educators who deal with assessment of their learners' diagnostic reasoning skills at the undergraduate and graduate medical education levels across disciplines.

To maximize small-group interaction and collaboration, the workshop required a room equipped with a projector and screen capable of displaying PowerPoint slides and videos, as well as an adequate sound system for attendees to hear spoken dialogue in the videos. An easel with a writing surface and markers were also used. Participants were placed into groups of five to six based on their seating position in the room, which was organized into small tables. An agenda and a facilitator guide (Appendices A and B) were created to ensure that the activities were presented in an orderly and timely manner. Participants were also supplied with worksheets to supplement the activities presented and allow for note-taking (Appendices C and D).

To meet the above learning objectives, the workshop included didactic teaching interspersed with role-playing, small-group activities, and practice with the ART. Facilitators began the workshop by introducing themselves and then asking for the participants to introduce themselves, including an icebreaking fun fact.

After introductions, the workshop followed the facilitator guide and agenda. A role-play activity (Appendix E) and reflection allowed participants to begin thinking of their own mental frameworks for diagnostic reasoning and helped facilitators to gauge the participants' prior knowledge in diagnostic reasoning concepts. Then, the didactic portion began with the PowerPoint presentation (Appendix F), which first described diagnostic errors and the general framework of diagnostic reasoning, followed by teaching in detail about each of the five domains of diagnostic reasoning using illustrative examples.

At this point, the didactic was paused to allow for an interactive exercise (Appendix C) in which participants were asked to work in pairs to create a problem representation statement with semantic qualifiers and then list their differential diagnoses. These were shared in the large group by volunteer participants, and semantic qualifiers from the statements were written on the easel by the facilitators to highlight as exemplars.

The didactic continued with a more detailed discussion of illness scripts followed by the resumption of the above activity (Appendix C), which asked the participants to create illness scripts based on their differential diagnoses.

The next section of the workshop included a discussion on cognitive biases and metacognition using an illustrated example case (Appendix D) and a large-group discussion on the types of biases displayed. The case used in the initial workshop was replaced with a shorter case after feedback from the evaluation forms (see below). The participants were guided towards additional training on each domain of the ART that could be reviewed through five brief online modules.[5]

In the final section of the workshop, the facilitators reintroduced the ART (Appendix G) and handed a paper version to participants to allow them to practice with the tool. Two video cases were shown, with the first case allowing groups to give their assessment and the second allowing groups to brainstorm feedback (Appendices H and I).

The workshop concluded with the facilitators asking for questions or comments from the participants on diagnostic reasoning, its domains, and the use of the ART.

For the workshop's adjustment to a 1-hour faculty development session (Appendix J), the bias activity (Appendix D) was removed. Some illustrative slides were also condensed. Otherwise, the workshop ran as noted above.

The workshop attendees at both the COMSEP 2019 annual meeting and the local institution were invited to complete an anonymous paper evaluation. The workshop evaluation tool

Copyright © 2020 Cohen et al. This is an open-access publication distributed under the terms of the Creative Commons Attribution license.

MedEdPORTAL® The AAMC Journal of Teaching and Learning Resources

featured 5-point Likert-scale items to obtain feedback about the workshop's ability to meet prespecified objectives, as well as open-ended questions soliciting qualitative comments about useful elements of the workshop and suggested improvements. After feedback, the form was revised to obtain more robust information on practice changes (Appendix K). Evaluation forms were collected and stored securely immediately after the sessions. No identifying information was collected on the evaluations.

Results

Nineteen participants attended the COMSEP 2019 annual meeting in the beginning of March 2019. Participants consisted of pediatrics faculty and clerkship directors, some of whom occupied educational leadership positions at their respective hospitals and medical schools. Our customized workshop evaluation was completed by 17 of the participants, who were asked to rate teaching effectiveness, the degree of achievement for each objective, and the usefulness of the session on a 5-point Likert scale (1 = *poor,* 5 = *excellent,* or 1 = *strongly disagree,* 5 = *strongly agree,* depending on the question). Participants were also asked if they intended to make a practice change. Likert-scale responses were analyzed with descriptive statistics to obtain the mean and standard deviation. Participants rated the facilitators and workshop favorably (Table 1), with means of 4.5 out of 5 or greater throughout the evaluation. Fifteen of the 17 participants noted they would make a change in their practice after the workshop, with one abstaining.

The workshop was conducted a second time at a large teaching hospital at the end of March 2019 with an estimated 15 participants. The faculty development session was geared towards pediatric hospital medicine physicians, although no demographic data were directly obtained. Eleven of the participants completed the same evaluation form, with similar results noted (Table 2). Nine of the participants indicated they would change their practice based on the workshop.

Participants were also asked for general narrative comments as well as specific comments about the most helpful aspects of the workshop and which parts they would change. Two authors (Adam Cohen and Satid Thammasitboon) reviewed the comments and analyzed them through iterative coding, clustering codes into like categories and developing themes. Of the 28 participants who evaluated the workshops, 19 (68%) left comments, which are summarized briefly by theme in Table 3.

Discussion

Our workshop was specifically designed to train clinical teachers on the concepts of diagnostic reasoning while preparing them to apply the theory-informed ART in their regular teaching practice. Overall, this workshop was well received at national and local levels by faculty of varying expertise, with modifications after each presentation based on participant feedback. Participants highlighted the benefits of using cases and video examples for interactive and hands-on activities. They reported that knowledge and skills gained from the sessions equipped them to apply the concepts in their teaching practice. Participants also appreciated the workshop's interactivity, which allowed for ample opportunity for discussion and learning from one another throughout the workshops.

Table 1. Summary of COMSEP Evaluations (*N* = 17)

Question	*M* (*SD*)
Facilitators[a]	
Speakers were clear about goals and objectives	4.7 (0.5)
Demonstrated sound communication skills	4.7 (0.5)
Facilitated interactive discussions/involved all participants	4.8 (0.4)
Listened and responded effectively to questions	4.8 (0.4)
Managed time well	4.7 (0.6)
Used effective tools to achieve objectives (handouts/slides, etc.)	4.8 (0.4)
Program objectives[a]	
Describe the cognitive processes in diagnostic reasoning	4.7 (0.6)
Define the five critical domains of diagnostic reasoning and their related components	4.6 (0.6)
Apply a novel instrument to the assessment and feedback of diagnostic reasoning in a medical learner	4.7 (0.5)
About this activity[b]	
I will use in my educational activities	4.5 (0.5)
Met my personal expectations	4.7 (0.5)
Updated my current knowledge	4.5 (0.6)

Abbreviation: COMSEP, Council on Medical Student Education in Pediatrics.
[a]Rated on a 5-point Likert scale (1 = *poor*, 3 = *good*, 5 = *excellent*).
[b]Rated on a 5-point Likert scale (1 = *strongly disagree*, 3 = *undecided*, 5 = *strongly agree*).

Table 2. Summary of Texas Children's Hospital Evaluations (*N* = 11)

Question	*M* (*SD*)
Facilitators[a]	
Speakers were clear about goals and objectives	4.6 (0.7)
Demonstrated sound communication skills	4.8 (0.4)
Facilitated interactive discussions/involved all participants	4.8 (0.4)
Listened and responded effectively to questions	4.8 (0.4)
Managed time well	4.7 (0.5)
Used effective tools to achieve objectives (handouts/slides, etc.)	4.7 (0.5)
Program objectives[a]	
Describe the cognitive processes in diagnostic reasoning	4.7 (0.5)
Define the five critical domains of diagnostic reasoning and their related components	4.6 (0.7)
Apply a novel instrument to the assessment and feedback of diagnostic reasoning in a medical learner	4.6 (0.5)
About this activity[b]	
I will use in my educational activities	4.7 (0.5)
Met my personal expectations	4.6 (0.5)
Updated my current knowledge	4.7 (0.5)

[a]Rated on a 5-point Likert scale (1 = *poor*, 3 = *good*, 5 = *excellent*).
[b]Rated on a 5-point Likert scale (1 = *strongly disagree*, 3 = *undecided*, 5 = *strongly agree*).

Copyright © 2020 Cohen et al. This is an open-access publication distributed under the terms of the Creative Commons Attribution license.

Table 3. Summary of Narrative Comments by Theme

Domain	Theme	Example Comments		
		General	Most Helpful	Additions/Changes
Workshop facilitators	The facilitators were knowledgeable and worked well together.	"Excellent team dynamic."<br>"All were well informed."		
Organization	The workshop was strategically organized and sequenced to facilitate learning.	"Well organized, good selection of topics promoting discussion."		
Interactive practice and discussion	The workshop was interactive, providing the opportunity for practice and discussion, which many marked as the most helpful aspect of the workshop.		"Interactive case exercise and video example."<br>"Interactive practicing at each step."<br>"Discussion at small group tables."	"Wish we could have done more cases."
The tool	Many also found the tool itself to be the most helpful part of the workshop.		"Tool for evaluation of diagnostic reasoning."<br>"The tool itself."	
Application of the tool	The application of the tool was seen as a positive learning opportunity in the workshop; however, many participants asked for additional information about how to apply the tool in their own practices and in different educational settings.		"Learned new tool and applied it."	"Still curious how to score and apply to promotion decisions."<br>"Use in remediation of struggling learners."<br>"Find an efficient way to incorporate portions of the tool into primary care peds practice."

As noted in the *Improving Diagnosis in Health Care* report, it is imperative that medical training programs enhance training of clinical reasoning for their learners in order to reduce the burden of diagnostic errors across all medical disciplines.[1] Currently, efforts at training learners in clinical reasoning are hampered by the lack of effective tools to assess learners' skill and deliver timely and formative feedback. A review of the literature indicated the availability of several assessment tools for diagnostic reasoning, but we found that the ART was unique in containing behavioral descriptors linked to various levels of performance in the major domains of the diagnostic reasoning process.[3,4,11-14] Apart from enhancing teachers' ability to assess their learners' diagnostic reasoning performance, the ART also offered shared construct and language that facilitated the delivery of feedback and aid in conation. The ART developers recognized the need for faculty training for optimal use of this tool and so created training modules for each domain of the ART (freely available online).[5] As we attempted to implement and validate the ART, our needs assessment dictated the necessity for more in-depth training for clinical teachers and faculty developers across disciplines regarding the concepts and shared terminology of diagnostic reasoning as well as the domains of the ART.

The ART developers had previously conducted several successful workshops on this topic locally and nationally. This workshop was the first time we employed an assessment for learning as a framework for the workshop. This framework allowed an effective interweaving between assessment domains (product) and theoretical concepts of diagnostic reasoning (process). Consequently, the workshop was highly interactive, was conducive for hands-on practice activities, and succinctly covered a large amount of content in a relatively short duration compared to our previous workshops. Even though the workshop was designed around the use of an assessment tool, we deliberately based its structure on a broader conceptual framework of diagnostic reasoning. This decision was made in order to allow participants with little to no knowledge of the diagnostic reasoning literature to quickly learn the necessary concepts in the use of the ART. This choice was reinforced by the evaluations, where a majority of participants reported that the workshop content was adequately covered and clearly explained.

We explicitly employed Kolb's experiential learning model[9,10] to structure the sequence of the workshop (Figure). This structure provided participants ample opportunities to bring in prior knowledge to establish learning goals, practice with new knowledge, experiment upon what they had learned, and immediately reflect on the experience. One of the highlights of the workshop was the use of video examples of case presentations for hands-on practice. Each video represented a different combination of behaviors that could be mapped to the ART domains. Participants were able to practice assessing these behaviors by giving feedback to peer participants. The participants were very engaged with the activity and received immediate feedback from other participants as well as for

Copyright © 2020 Cohen et al. This is an open-access publication distributed under the terms of the Creative Commons Attribution license.

the workshop facilitators through think-pair-share and group discussions, respectively. The workshop evaluations indicated an overwhelmingly positive response to this structural aspect of the workshop, with multiple comments mentioning how the interactive practice using expert guidance of the facilitators was the most helpful part of the workshop.

We utilized the information garnered from our first workshop to implement a longitudinal assessment practice at our institution where hospitalist faculty could utilize the ART as an assessment as well as a feedback tool for residents during their rotation. These faculty used the tool after presentations of new admissions to give residents feedback on their reasoning.

We also had the following observations and reflections after our experiences facilitating this workshop. We noted that audience expertise in the field of diagnostic reasoning could be markedly variable, especially in the use of terminology such as illness scripts and semantic qualifiers. We recognized that the duration of time spent on the introductory segment of workshop might need to be rapidly adjusted after preliminary assessment of participants' backgrounds and experience. Furthermore, while the ART was accepted by participants as a valuable tool to assess learners and give them feedback on diagnostic reasoning, it still required practice to attain familiarity with the behavioral descriptors linked to various levels of performance. Hence, during the workshop, it is important to emphasize the need for repeated use of the ART to minimize dissatisfaction and promote its adoption. For the same reason, it is important to balance the need to play the whole video without interruption to emulate an authentic clinical situation versus pausing the video after the learner presents each domain so that participants can focus on each domain specifically. We found that using the initial practice video to allow for pauses and playing the second video through struck that balance well. Additionally, some participants struggled with the prompt of metacognition in the videos as they wondered if this was a practical or accurate representation of the learner's actual practice. Facilitators need to emphasize that metacognition is an advanced skill. Prompting learners to reflect upon their own cognitive tendencies and emotional factors is an initial step for a reflective practice.

With regard to the ART and the feedback process, while the ART helps deliver focused and concrete feedback, the process of diagnostic reasoning tends to be very idiosyncratic, and thus, giving feedback on this process remains a crucial yet difficult task. We recognize the need for timely feedback, but questions remain about the right time and setting for the supervising physician to deliver this feedback to the learner. The process of feedback is inherently complicated, which is why it is so important to allow more time for this crucial component at the end of the workshop so participants can delve deeper into reflection and discussion of the use of ART as a tool to guide feedback.

Although pediatric providers have been the main audience for the workshop so far, the favorable responses and positive feedback from providers within a variety of different educational roles and subspecialties suggest the workshop's generalizability. Furthermore, our workshop facilitators were from various specialties, and all had a favorable response to the workshop. Finally, as-yet unpublished results of the study that validated the ART consisted of educators across multiple disciplines and institutions.

There are recognized limitations to this work. First, our evaluation tool mainly measured the reaction of the participants to the workshop. While this is the lower tier of the Kirkpatrick's model, our results still demonstrate the feasibility of this intervention to audiences of varying expertise and disciplines. Second, our workshop is based on one conceptual framework of diagnostic reasoning, and while it fits with the ART, other frameworks may resonate differently with different populations of physicians. Finally, all of the facilitators of this workshop had prior knowledge of the concepts of diagnostic reasoning that were outlined. Although only one of the facilitators had involvement in the development of the conceptual frameworks and the ART, this does suggest that other facilitators will need to review the concepts prior to presenting the workshop.

Our next step is to continue to improve this workshop to guide the development of diagnostic reasoning expertise in accordance with Association of Medical Colleges Entrustable Professional Activities (EPAs) and Accreditation Council of Graduate Medical Education milestones. As medical education continues to move towards competency-based assessment, the core EPAs for entering residency and the residency milestones are at the heart of the competencies.[15,16] As the ART is based on specific competencies of clinical reasoning, it overlaps with multiple EPAs and milestones and may lend itself to being a useful tool in the evaluation of medical students and residents.

Overall, this faculty development workshop and its materials represent an important step in the workplace-based assessment of diagnostic reasoning in medical learners. The workshop is grounded in the clinical reasoning literature and presents one approach to assessing these skills in learners with or without direct observation of clinical skills. The workshop can continue

Copyright © 2020 Cohen et al. This is an open-access publication distributed under the terms of the Creative Commons Attribution license.

to be evaluated in a varied population of medical educators to assess for changes in knowledge and behavior.

Appendices

A. Workshop Agenda.docx
B. Facilitator Guide.docx
C. Problem Representation Exercise.docx
D. Cognitive Bias Exercise.docx
E. Diagnostic Reasoning Role-Play.docx
F. PowerPoint Presentation for 2-Hour Workshop.pptx
G. Assessment of Reasoning Tool.pdf
H. Live Example - Poor Diagnostic Reasoning.mov
I. Live Example - Excellent Diagnostic Reasoning.mov
J. PowerPoint Presentation for 1-Hour Workshop.pptx
K. Ideal Evaluation Form.docx

All appendices are peer reviewed as integral parts of the Original Publication.

Adam Cohen, MD: Assistant Professor, Section of Hospital Medicine, Department of Pediatrics, Baylor College of Medicine/Texas Children's Hospital; ORCID: https://orcid.org/0000-0003-4453-3792

Moushumi Sur, MD: Associate Professor, Section of Critical Care Medicine, Department of Pediatrics, Baylor College of Medicine/Texas Children's Hospital

Martin Weisse, MD: Professor, Section of Infectious Disease, Department of Pediatrics, West Virginia University School of Medicine

Kathryn Moffett, MD: Professor, Section of Infectious Disease, Department of Pediatrics, West Virginia University School of Medicine

Jeffrey Lancaster, MD: Associate Professor, Section of General Pediatrics, Department of Pediatrics, West Virginia University School of Medicine

Renee Saggio, MD: Professor, Section of General Pediatrics, Department of Pediatrics, West Virginia University

Geeta Singhal, MD, MEd: Professor, Section of Hospital Medicine, Department of Pediatrics, Baylor College of Medicine/Texas Children's Hospital

Satid Thammasitboon, MD, MHPE: Associate Professor, Section of Critical Care Medicine, Center for Research, Innovation and Scholarship in Medical Education, Department of Pediatrics, Baylor College of Medicine/Texas Children's Hospital

Disclosures

None to report.

Funding/Support

None to report.

Ethical Approval

Reported as not applicable.

References

1. National Academies of Sciences, Engineering, and Medicine. *Improving Diagnosis in Health Care*. National Academies Press; 2015:355-402.
2. Gruppen LD. Clinical reasoning: defining it, teaching it, assessing it, studying it. *West J Emerg Med*. 2017;18(1):4-7. https://doi.org/10.5811/westjem.2016.11.33191
3. Thammasitboon S, Rencic JJ, Trowbridge RL, Olson APJ, Sur M, Dhaliwal G. The Assessment of Reasoning Tool (ART): structuring the conversation between teachers and learners. *Diagnosis (Berl)*. 2018;5(4):197-203. https://doi.org/10.1515/dx-2018-0052
4. Daniel M, Rencic J, Durning SJ, et al. Clinical reasoning assessment methods: a scoping review and practical guidance. *Acad Med*. 2019;94(6):902-912. https://doi.org/10.1097/ACM.0000000000002618
5. Assessment of Reasoning Tool. Society to Improve Diagnosis in Medicine. 2019. Accessed May 7, 2019. https://www.improvediagnosis.org/art/
6. Pitre C. Diagnostic reasoning assessment toolkit: guided reflection and standardized cases for at-risk final-year medical students. *MedEdPORTAL*. 2016;12:10428. https://doi.org/10.15766/mep_2374-8265.10428
7. Lane K, Rhodes M, Olson A. Diagnostic reasoning feedback: improving diagnostic reasoning skills and feedback satisfaction through structured peer feedback. *MedEdPORTAL*. 2016;12:10340. https://doi.org/10.15766/mep_2374-8265.10340
8. Packard K, Cochran T, Huggett K, et al. The interprofessional team reasoning framework. *MedEdPORTAL*. 2013;9:9460. https://doi.org/10.15766/mep_2374-8265.9460
9. Kolb DA. *Experiential Learning: Experience as the Source of Learning and Development*. 2nd ed. Pearson Education; 2015.
10. Armstrong E, Parsa-Parsi R. How can physicians' learning styles drive educational planning? *Acad Med*. 2005;80(7):680-684. https://doi.org/10.1097/00001888-200507000-00013
11. Norcini JJ, Blank LL, Duffy FD, Fortna GS. The mini-CEX: a method for assessing clinical skills. *Ann Intern Med*. 2003;138(6):476-481. https://doi.org/10.7326/0003-4819-138-6-200303180-00012
12. Abouna GM. The Integrated Direct Observation Clinical Encounter Examination (IDOCEE)-an objective assessment of students' clinical competence in a problem-based learning

Copyright © 2020 Cohen et al. This is an open-access publication distributed under the terms of the Creative Commons Attribution license.

curriculum. *Med Teach*. 1999;21(1):67-72. https://doi.org/10.1080/01421599980066

13. Carter C, Akar-Ghibril N, Sestokas J, Dixon G, Bradford W, Ottolini M. Problem representation, background evidence, analysis, recommendation: an oral case presentation tool to promote diagnostic reasoning. *Acad Pediatr*. 2018;18(2): 228-230. https://doi.org/10.1016/j.acap.2017.08.002

14. van der Vleten CPM, Norman GR, Schuwirth LWT. Assessing clinical reasoning. In: Higgs J, Jones M, Loftus S, Christensen N, eds. *Clinical Reasoning in the Health Professions*. 3rd ed. Elsevier, Churchill Livingstone; 2008:413-421.

15. The core Entrustable Professional Activities (EPAs) for entering residency. *Association of American Medical Colleges*. May 2014. Accessed May 14, 2019. https://www.aamc.org/initiatives/coreepas/

16. Milestones. Accreditation Council for Graduate Medical Education. Accessed May 14, 2019. https://www.acgme.org/What-We-Do/Accreditation/Milestones/Overview

Received: August 2, 2019
Accepted: January 24, 2020
Published: August 21, 2020

Copyright © 2020 Cohen et al. This is an open-access publication distributed under the terms of the Creative Commons Attribution license.
