# Supplementary material for: Teaching Diagnostic Reasoning to Faculty Using an Assessment for Learning Tool: Training the Trainer
Source: MedEdPORTAL. 2020 Aug 21;16:10938. doi: 10.15766/mep_2374-8265.10938 (PMC7449581; doi:10.15766/mep_2374-8265.10938)
Supplement: Supplementary file 1 — Workshop Agenda.docxFacilitator Guide.docxProblem Representation Exercise.docxCognitive Bias Exercise.docxDiagnostic Reasoning Role-Play.docxPowerPoint Presentation for 2-Hour Workshop.pptxAssessment of Reasoning Tool.pdfLive Example - Poor Diagnostic Reasoning.movLive Example - Excellent Diagnostic Reasoning.movPowerPoint Presentation for 1-Hour Workshop.pptxIdeal Evaluation Form.docx [file mep_2374-8265.10938-s001.zip › A. Workshop Agenda.docx]

**Appendix A: Workshop Agenda**

| **ACTIVITY** | **TIME** |
| --- | --- |
| Introductions | 5 mins |
| Diagnostic reasoning process-Role play and Facilitated group discussion  (<5 min role play, 5 quick general reflection, do not go deep into domains of reasoning) | 10 mins |
| What is inside the Black Box?  (How do we define what characteristics of an oral case presentation are good or not pertaining to diagnostic reasoning? Personal experiences and Learners observations) | 10 min |
| Didactic: SIDM Conceptual Framework, Hypothesis-directed, features | 10 mins |
| Didactic: Problem representation, Semantic Q, IS | 5 mins |
| Paired exercises (PR and IS)  (Introduce exercise and lead large group report and discussion) | 10 mins |
| Didactic: Create & compile IS towards expertise | 5 min |
| Introduce the cognitive biases (slide and refer to handout of Pectus case, one participant reads out loud)  Lead large group discussion “What happened?” (about cognitive tendencies, emotional and situational factors, and introduce metacognition) | 10 min |
| Reintroduce the ART and rate 1^st^ video  In large group, rate the video, one domain at a time | 10 min |
| Introduce 2^nd^ Video and Feedback Framework  Watch from start to finish, In small groups, rate the video and practice/discuss in what/how to give ART-guided feedback  Facilitated group discussion | 15 min |
| Take home message | 5 mins |
| TOTAL | 95 minutes |

*Boxes in gray were omitted or significantly shortened in 1 hour version of workshop
