# Supplementary material for: Teaching Diagnostic Reasoning to Faculty Using an Assessment for Learning Tool: Training the Trainer
Source: MedEdPORTAL. 2020 Aug 21;16:10938. doi: 10.15766/mep_2374-8265.10938 (PMC7449581; doi:10.15766/mep_2374-8265.10938)
Supplement: Supplementary file 1 — Workshop Agenda.docxFacilitator Guide.docxProblem Representation Exercise.docxCognitive Bias Exercise.docxDiagnostic Reasoning Role-Play.docxPowerPoint Presentation for 2-Hour Workshop.pptxAssessment of Reasoning Tool.pdfLive Example - Poor Diagnostic Reasoning.movLive Example - Excellent Diagnostic Reasoning.movPowerPoint Presentation for 1-Hour Workshop.pptxIdeal Evaluation Form.docx [file mep_2374-8265.10938-s001.zip › B. Facilitator Guide.docx]

Facilitator’s Guide

1. **How to Conduct a Role Play and Facilitate a Large Group Discussion:**

The script for this role play reflects an oral presentation of a patient encounter by a clinical learner who has not attained proficiency in the domains of clinical reasoning. The learner presents many irrelevant details, omits or glosses over important information during his analysis, appears to lack the ability to synthesize the data in a cohesive manner, and does not organize the information to reflect his or her diagnostic reasoning. The facilitators may enact the role play themselves or request participants to volunteer. This role play does not require advance preparation as volunteers may simply read from the script.

The facilitator may choose to modify the specifics of the case to appear relevant to the participants depending upon their specialties. However, based on our experience, the case does not necessarily have to align with the clinical specialty of the workshop participants. The benefit of using a case that does not fit the participants’ content area (e.g. a pediatric case for internal medicine participants) is that the focus of the discussion will be on the demonstration of diagnostic reasoning skills and not the specific details of the case.

After the role play, the facilitator should invite a large group discussion by asking the following questions:

- What are your thoughts about this learner’s presentation?
- What are some considerations that help you to determine whether this is a good, average or poor oral presentation?
- How do you come to a conclusion regarding competency of this learner’s clinical reasoning skills?

The moderated discussion allows participants articulate their prior knowledge or idiosyncratic criteria that they may have used to assess the diagnostic reasoning skills of their learners. The facilitator should avoid a prolonged discussion about each participant’s specific idiosyncratic approach to assessing diagnostic reasoning skills or do a deep dive into the intricacies of such an assessment.

2. **Concepts and Terminologies used in the Didactic Presentation**: Domains of diagnostic reasoning according to the Assessment of Reasoning Tool
   a. **Hypothesis-directed data gathering**: After initial open-ended questioning, data collection is clearly linked to specific diagnoses, aiming to increase or decrease the likelihood of these diagnoses rather than just asking a non-directed list of questions.
      i. **Defining Features:** descriptors that are characteristic of the diagnoses
      ii. **Discriminating Features:** descriptors that are useful for distinguishing the diagnoses from one another
   b. **Problem representation**: A concise statement that translates the key patient data into medical terminology in a synthesized format reflecting “mental representation” about the case. The problem representation usually includes basic epidemiology (57-year-old man), relevant (but not all) co-morbidities, time course (both onset and temporal relationship), and key clinical findings (e.g. defining and discriminating features). A good problem representation must not include distractors and non-pertinent data.
      i. **Semantic Qualifiers:** Specific terms that categorize patient presentations into clear and recognizable patterns and facilitate differentiation between potential diagnoses. Semantic qualifiers often imply opposing qualities within the term. Examples include acute/sub-acute/chronic, intermittent/progressive, and localized/diffuse.
      ii. **Illness scripts:** Abstract representations of diseases. A commonly used construct for an illness script is a three-component illness script: epidemiology (age, underlying characteristics or conditions); temporal relationship (onset, duration, progress), and syndrome (key signs/symptoms)
   c. **Prioritized differential diagnosis**: An articulation of diagnostic possibilities listed in descending order as ranked by the degree of matching with the problem representation. The following is one of many proposed structures:
      i. Tier I: diagnoses that are near perfect match with the problem representation
      ii. Tier Ie: emergent and highly morbid diagnosis that may have variable match with the problem representation
      iii. Tier II: diagnoses that partially match the problem representation
      iv. Tier III: diagnoses with match the problem representation only to a limited degree
   d. **Direct evaluation towards leading diagnostic hypotheses** directed toward Tier I and sometimes Tier Ie diagnoses; less often tier II, and rarely tier III; overall testing and treatment embodies high value care.
   e. **Metacognition**– Analyzing one’s own thought processes and emotional state and recognizing potential predispositions to error. Being able to answer the following question: Is there anything about the way you are thinking or feeling about this case that may lead to error? These may include, but are not limited to, cognitive tendencies, emotional/situational factors such as the following:

*Cognitive Tendencies*

- **Availability Heuristic** – accepting a diagnosis due to ease in recalling similar event or case rather than based on probability
- **Representative Heuristic** – Improper use of pattern recognition to detect a ‘prototype’ to diagnose a condition
- **Anchoring** – Fixate on specific features of a presentation too early in diagnostic process, and stay with an original diagnosis despite evidence to the contrary
- **Ascertainment** – to see only what one hopes to find
- **Confirmation** – Looing for only confirming evidence of the hypothesis rather than disconfirming evidence to dispute it
- **Diagnosis Momentum** – an opinion or working diagnosis becomes almost certain when it is passed from person to person, suppressing further evaluation
- **Omission** – watchful waiting for fear of being held responsible for adverse outcomes
- **Premature Closure** – apply closure to a diagnosis too early so that the correct diagnosis is not considered

*Affective Biases*

- Team Factors – team dynamic that can increase risk of misdiagnosis
- Fatigue/Sleep Deprivation
- Emotional Factors – emotional attachment, compassion fatigue
- Patient Factors – the care provider’s response to various patient factors (e.g. age, gender, race)

The content above is adapted from the work by the Society to Improve Diagnosis (SIDM) Assessment Subcommittee. The facilitator should review the faculty training modules on the SIDM Website. The facilitator should also encourage workshop participants to review prior knowledge and acquire further knowledge by viewing these modules.

3. **Assessing a learner using the Assessment of Reasoning Tool (ART)**

After the brief didactic session introducing the Assessment of Reasoning Tool to the participants, the facilitator should instruct participants to attempt hands-on practice using the ART to assess a learner. As the ART comprises five domains, the facilitator may choose to deconstruct the ART by presenting each domain of the ART with clarifying concepts described above, one by one. This approach should be considered for novice participants.

The hands-on practice uses a short video demonstrating a learner presenting a case to a supervising physician. The facilitator should allow participants to review the ART prior to viewing the video. The facilitators may choose one of the two options to facilitate this practice.

a. Show the video from the start to finish, and then instruct participants to use the ART to assess the learner’s performance
b. Show the video with pauses and instruct participants to assess one domain at a time

We have found Option b to be well received by novice participants, although this option is not as authentic as Option a. If the participants have already viewed the online faculty training modules prior to the workshop, Option a is preferred. If there is ample time (a 2-hour workshop), we recommend a mixed approach as following:

The video demonstrating a learner presenting a case summary to his supervising physician outside a patient room is played on a large screen. The video is first played uninterrupted. Next, the video is replayed with pauses at the end of each domain (i.e. the video is paused at the end of the learner’s data gathering presentation, then after his problem representation, then after he presents his differential diagnosis, then after his presentation of diagnostic tests needed and lastly, after his demonstration of prompted metacognition).

We created two videos (in Appendix F and J) of a learner discussing the case of a teenage female presenting with abdominal pain and dysuria who is sexually active. The reason for this careful case selection was to choose a case that would be broadly applicable and relevant to multiple subspecialties. We believed that the case we chose could present in multiple settings including an emergency department, clinic or inpatient hospital environment, and could be evaluated by a surgeon, an internist, a pediatrician, a family practitioner or even a gynecologist.

In this first video, the learner displays the ability to do a complete hypothesis-directed history and physical exam as per his oral presentation to the supervising physician. He also formulates a complete problem representation using accurate semantic qualifiers, a complete yet prioritized differential diagnosis and a high value diagnostic evaluation. After his case summary, the supervising physician asks whether the learner has considered emotional or cognitive factors influencing his reasoning for the case. This prompts the learner to demonstrate his metacognitive abilities in reflecting upon and verbalizing his cognitive tendencies and/or emotional factors, of which he has little insight.

If the facilitators choose, they can replace the robotic videos in Appendix F and J with the live videos in Appendix H and I. Appendix I should replace this first video, and reflects the same presentation as above, except the participant displays excellent metacognitive skills.

After participants have watched the video, they then use the ART to assess the learner oral case presentation. Some participants might need additional explanations of the concepts and terms used in the ART, so a team of facilitators should walk around the room to assist participants at each table during this practice session.

Facilitator should allocate sufficient time for a large group discussion after the exercise. The discussion should also allow the reflection on the exercise, addressing strengths and weaknesses of the ART and sharing practical tips effective use of the ART. The facilitator should moderate the discussion about how ones may use the ART in different settings.

4. **Giving feedback to a learner based on the Assessment of Reasoning Tool (ART)**

This hands-on practice should follow the same steps as the first (assessing a learner) practice session. The second video using the same case of abdominal pain and dysuria in a sexually active teenage girl is displayed to the participants. However, the learner in this second video demonstrates varying abilities for each diagnostic reasoning domain. He shows minimal hypothesis direction in his data gathering, his problem representation is incomplete with no semantic qualifiers used, his differential diagnosis and diagnostic evaluation plan is partially complete, and when prompted by the supervising physician, he demonstrates excellent metacognitive in verbalizing potential signs of bias.

The participants are instructed to assess the second learner’s presentation and use the ART again to frame their feedback to this learner.

If the facilitators choose, they can replace the robotic videos with the live videos in appendix H and I. Appendix H should replace this first video, and reflects the same presentation as above, except the participant displays poor metacognitive skills.
