# Supplementary material for: Teaching Diagnostic Reasoning to Faculty Using an Assessment for Learning Tool: Training the Trainer
Source: MedEdPORTAL. 2020 Aug 21;16:10938. doi: 10.15766/mep_2374-8265.10938 (PMC7449581; doi:10.15766/mep_2374-8265.10938)
Supplement: Supplementary file 1 — Workshop Agenda.docxFacilitator Guide.docxProblem Representation Exercise.docxCognitive Bias Exercise.docxDiagnostic Reasoning Role-Play.docxPowerPoint Presentation for 2-Hour Workshop.pptxAssessment of Reasoning Tool.pdfLive Example - Poor Diagnostic Reasoning.movLive Example - Excellent Diagnostic Reasoning.movPowerPoint Presentation for 1-Hour Workshop.pptxIdeal Evaluation Form.docx [file mep_2374-8265.10938-s001.zip › C. Problem Representation Exercise.docx]

Case Scenario

A 2-week-old female infant presents to the pediatric clinic with a chief complaint of vomiting for 1 day.

She has been fine until 4 days ago when she was not acting normally. She has had decreased appetite for 3 days. Since last night, she has been vomiting every time she eats. The vomiting is projectile with small amount of secretion or formula after feeding. Mom says the baby has had intermittent spitting since birth, and has been told by her PCP that she has reflux. She has no diarrhea, no bloody stool, no fever, no congestion and no sick contact. Mom notices she is sleeping a lot today.

She was born full term, NSVD, BW 3,200 g, GBS-negative, passed meconium on the first day

PE: Temp 36 °C, PR 180, RR 55, BP 60/40, SpO_2_ 95%

GA: toxic looking, pale, good skin turgor

HEET: dry lips, moist membranes, flat fontanelle, normocephalic, atraumatic

Respiratory: mild distress, mild retractions, no rales, no wheezing

Cardiovascular: normal S_1_S_2_, no murmur, cap refill 4 sec, cool feet

Abdomen: diminished bowel sounds, no distension, no hepatosplenomegaly, no mass, no tenderness

Neuro: lethargic, grossly intact cranial nerves, normal DTRs, no clonus, no stiff neck, negative kernig & brudzinski.

**Problem representation:** Synthesize the case using semantic qualifiers in 1-2 sentences to capture the ‘big picture’

**DDX:** list 3 most likely diagnoses based on your problem representation.

1. ________________
2. ________________
3. ________________

Compare and Contrast Illness Scripts

| **Script No.1** | **Script No.2** |
| --- | --- |
|  |  |
|  |  |
|  |  |
|  |  |
|  |  |
|  |  |
| **Diagnosis 1:** | **Diagnosis 2:** |
