# Supplementary material for: Teaching Diagnostic Reasoning to Faculty Using an Assessment for Learning Tool: Training the Trainer
Source: MedEdPORTAL. 2020 Aug 21;16:10938. doi: 10.15766/mep_2374-8265.10938 (PMC7449581; doi:10.15766/mep_2374-8265.10938)
Supplement: Supplementary file 1 — Workshop Agenda.docxFacilitator Guide.docxProblem Representation Exercise.docxCognitive Bias Exercise.docxDiagnostic Reasoning Role-Play.docxPowerPoint Presentation for 2-Hour Workshop.pptxAssessment of Reasoning Tool.pdfLive Example - Poor Diagnostic Reasoning.movLive Example - Excellent Diagnostic Reasoning.movPowerPoint Presentation for 1-Hour Workshop.pptxIdeal Evaluation Form.docx [file mep_2374-8265.10938-s001.zip › D. Cognitive Bias Exercise.docx]

**CASE 3**

**History**

- 4-year-old child presented to the emergency room (ER) of a tertiary care hospital with low grade fever, tachypnea and hypoxemia.
- Fairly typical and busy upper respiratory winter season.
- Child was noted to be tachypneic and in mild respiratory distress.
- On chest exam, rales and wheezes heard on the right side.

**Diagnostic Tests**

- Chest radiograph revealed right sided air space disease with a moderately large same-side pleural effusion and mildly enlarged heart size.
- Chest ultrasound confirmed a large unloculated pleural effusion.

**Plan in the ER**

- Surgery consultation obtained in the ER recommended a video-assisted thorascopic surgery (VATS) in the operating room next day.
- To evaluate the cardiac size, the ER resident ordered a BNP. However, the results were not followed up prior to transfer to the inpatient setting.

**Inpatient Course**

- The admitting team noted the child to be “tachypneic but stable” and planned to continue empiric antibiotics.
- On rounds the next morning, child was found to be “restless, tachypneic, and with an increasing oxygen requirement.”
- BNP reported as 4149 pg/ml. Informal discussion with cardiology fellow suggested that the elevated BNP could be due to the respiratory illness itself.
- Due to scheduling delays in the operating room, the treating physicians decided to pursue an ultrasound -guided thoracentesis.

The interventional radiologist aspirated a clear transudate suggestive of congestive heart failure. Cardiologist was called to bedside for a stat echocardiogram that revealed a dilated heart with an EF of 28%.

**Final Outcome**

- Child diagnosed with a viral myocarditis and was in early congestive heart failure.

**CASE 3: Cognitive Autopsy**

- First, the child presented with pneumonia- like symptoms (in peak winter season) to a tertiary care ER where many similar cases are routine—this created an “**availability heuristic**.”
- Second, ER physicians created a **“framing effect**” when they “packaged” the patient as a definite “pneumonia/rule out empyema” to the admitting team and surgeon.
- Once this diagnosis was “framed” as pneumonia, the admitting team did not pursue competing diagnoses, even though several clues to an alternative diagnosis were present.
- The third cognitive problem in this case was a “**confirmation bias**” of the admitting team, who demonstrated a tendency to look for proof of the pneumonia and empyema while ignoring factors such as the elevated BNP and cardiomegaly. The BNP in this case was remarkably high and suggested heart failure.
- Lastly, the admitting team had 15 patients to round on that day and was overwhelmed; in addition, the resident assigned to care for this patient was post-call. The time pressures surrounding the care of the patient and other patients may have led to “**premature closure**.”
