# Supplementary material for: Teaching Diagnostic Reasoning to Faculty Using an Assessment for Learning Tool: Training the Trainer
Source: MedEdPORTAL. 2020 Aug 21;16:10938. doi: 10.15766/mep_2374-8265.10938 (PMC7449581; doi:10.15766/mep_2374-8265.10938)
Supplement: Supplementary file 1 — Workshop Agenda.docxFacilitator Guide.docxProblem Representation Exercise.docxCognitive Bias Exercise.docxDiagnostic Reasoning Role-Play.docxPowerPoint Presentation for 2-Hour Workshop.pptxAssessment of Reasoning Tool.pdfLive Example - Poor Diagnostic Reasoning.movLive Example - Excellent Diagnostic Reasoning.movPowerPoint Presentation for 1-Hour Workshop.pptxIdeal Evaluation Form.docx [file mep_2374-8265.10938-s001.zip › E. Diagnostic Reasoning Role-Play.docx]

**Teaching Diagnostic Reasoning Using an Assessment for Learning Tool: A Train-the-Trainer Session**

**Attending**: So, are you ready to tell me about our patient in room 3?

**Student**: Yes. Little Lily is a 2-week-old girl who is presenting today because of new vomiting. She was doing well until 4 days ago when Mom noted she wasn’t acting right and sleeping more. She began to refuse breast feeding, eating once every 4-6 hours instead of every 2-3 hours. Last night she started having vomiting as well. The vomiting looks like formula after feeding, but is forceful per the parents. She has had some spit ups since birth, and has been diagnosed with reflux, but this feels different to the parents. They do not report any other symptoms.

Lets see….past medical history, I mentioned she was full term. She has other wise been healthy. No surgeries, medications, or allergies. In terms of family history, the maternal grandmother has a history of breast cancer and the paternal uncle has Crohn’s disease. In term of social history, she lives with her mother and father, and they have a brown lab.

On exam, she was afebrile, pulse was 180, respiratory rate was 54, blood pressure was 60/40, and saturations were 95%. She looked pale and weak. Her fontanelle seemed flat. She had dry lips but moist mucous membranes. I couldn’t see her tonsils or her tympanic membranes. In terms of lung exam, she had mild subcostal retractions, but sounded clear. Her heart sounded normal with no murmur, but her cap refill was 4 seconds and her feet felt cool. Her belly was soft and non-tenders. I didn’t note distention, hepatomegaly or masses. She was not really responding very well or playful, but she could move all of her extremities. She did not have a stiff neck.

*Student looks up at attending for approval*

**Attending:** So, what do you think is going on?

**Student**: In summary, this is a 2-week-old ex-FT previously healthy infant who presents with acute onset NBNB forceful vomiting, who is sick appearing, tachycardic and in some respiratory distress. The differential can be broad, including infectious vomiting, worsening of underlying reflux, or obstruction. I definitely wouldn’t want to miss obstruction, and it may be more likely, because the vomiting is projectile. It could be from pyloric stenosis, which she is the right age for, but I would expect her to have a palpable olive on exam. It could be a deeper obstruction like volvulus, but I would expect bilious emesis.

**Attending:** What would you like to do for her?

**Student**: Since she looks dehydrated, I thought we could start some IV fluids, and maybe get an ultrasound to look for pyloric stenosis.

**Attending**: Is there anything about the way you are thinking or feeling about this case that may lead to error?

**Student:** I don’t think so. I mean she’s just a baby.
