# Supplementary material for: Teaching Diagnostic Reasoning to Faculty Using an Assessment for Learning Tool: Training the Trainer
Source: MedEdPORTAL. 2020 Aug 21;16:10938. doi: 10.15766/mep_2374-8265.10938 (PMC7449581; doi:10.15766/mep_2374-8265.10938)
Supplement: Supplementary file 1 — Workshop Agenda.docxFacilitator Guide.docxProblem Representation Exercise.docxCognitive Bias Exercise.docxDiagnostic Reasoning Role-Play.docxPowerPoint Presentation for 2-Hour Workshop.pptxAssessment of Reasoning Tool.pdfLive Example - Poor Diagnostic Reasoning.movLive Example - Excellent Diagnostic Reasoning.movPowerPoint Presentation for 1-Hour Workshop.pptxIdeal Evaluation Form.docx [file mep_2374-8265.10938-s001.zip › G. Assessment of Reasoning Tool.pdf]

ASSESSMENT of REASONING TOOL

SOCIETY to IMPROVE DIAGNOSIS in MEDICINE

Learner: ______________________ Evaluator: ______________________

Did the Learner…	Assessment		
	Minimal	Partial	Complete
Collect/report history and examination data in a **hypothesis-directed manner**?	• Non-directed in questioning and exam<br>• Asked questions without clear focus on potential diagnoses	• Questioning and exam generally reflective of potential diagnoses, but some less relevant or tangential questions	• Followed clear line of inquiry, directing questioning and exam to specific findings likely to increase or decrease likelihood of specific diagnoses
Articulate a complete **problem representation** using descriptive medical terminology?	• Included extraneous information<br>• Missed key findings<br>• Did not translate findings into medical terminology	• Generally included key clinical findings (both positive and negative) but either missed some key findings or missed important descriptive medical terminology	• Gave clear synopsis of clinical problem<br>• Emphasized important positive and negative findings using descriptive medical terminology
Articulate a **prioritized differential diagnosis** of most likely, less likely, unlikely, and "can't miss" diagnoses based on the problem representation?	• Missed key elements of differential diagnosis, including likely diagnoses or "can't miss" diagnoses	• Gave differential diagnosis that included likely and "can't miss" diagnoses but either missed key diagnoses or ranked them inappropriately	• Gave accurately ranked differential diagnosis including likely and "can't miss" diagnoses
Direct evaluation/treatment towards **high priority diagnoses**?	• Directed evaluation and treatment toward unlikely/unimportant diagnoses<br>• Did not evaluate or treat for most likely/"can't miss" diagnoses	• Major focus of evaluation and treatment was likely and "can't miss" diagnoses but included non-essential testing	• Efficiently directed evaluation and treatment towards most likely and "can't miss" diagnoses<br>• Deferred tests directed towards less likely or less important diagnoses
Demonstrate the ability to **think about their own thinking** (metacognition)?<br>Consider asking: *Is there anything about the way you are thinking or feeling about this case that may lead to error?*	• Not able to describe the influence of cognitive tendencies or emotional/situational factors that may have influenced decision-making	• Can name one cognitive tendency or emotional/situational factor that may have influenced decision-making	
**OVERALL ASSESSMENT**	NEEDS IMPROVEMENT ☐	MEETS COMPETENCY ☐	EXCELLENCE ☐

Comments:
