# Supplementary material for: Teaching Diagnostic Reasoning to Faculty Using an Assessment for Learning Tool: Training the Trainer
Source: MedEdPORTAL. 2020 Aug 21;16:10938. doi: 10.15766/mep_2374-8265.10938 (PMC7449581; doi:10.15766/mep_2374-8265.10938)
Supplement: Supplementary file 1 — Workshop Agenda.docxFacilitator Guide.docxProblem Representation Exercise.docxCognitive Bias Exercise.docxDiagnostic Reasoning Role-Play.docxPowerPoint Presentation for 2-Hour Workshop.pptxAssessment of Reasoning Tool.pdfLive Example - Poor Diagnostic Reasoning.movLive Example - Excellent Diagnostic Reasoning.movPowerPoint Presentation for 1-Hour Workshop.pptxIdeal Evaluation Form.docx [file mep_2374-8265.10938-s001.zip › K. Ideal Evaluation Form.docx]

##### WORKSHOP EVALUATION

| **Today’s Date:** | **Teaching Diagnostic Reasoning using an Assessment for Learning Tool- A Train-the-Trainer Session** | |
| --- | --- | --- |
| **Facilitators:** | |  |

| FACILITATORS  Please indicate the overall teaching effectiveness of the facilitators by circling ONE number. | Excellent | **Very Good** | Good | Fair | Poor |
| --- | --- | --- | --- | --- | --- |
| 1. Speakers were clear about goals and objectives | 5 | 4 | 3 | 2 | 1 |
| 2. Demonstrated sound communication skills | 5 | 4 | 3 | 2 | 1 |
| 3. Facilitated interactive discussions/involved all participants | 5 | 4 | 3 | 2 | 1 |
| 4. Listened and responded effectively to questions | 5 | 4 | 3 | 2 | 1 |
| 5. Managed time well | 5 | 4 | 3 | 2 | 1 |
| 6. Used effective tools to achieve objectives (handouts/slides etc) | 5 | 4 | 3 | 2 | 1 |

**Comments:**

| PROGRAM OBJECTIVES  Please indicate how well each of the stated program objectives was ACHIEVED by circling ONE number for EACH item.  **At the conclusion of the activity, the participants should be able to:** | Excellent | **Very Good** | Good | Fair | Poor |
| --- | --- | --- | --- | --- | --- |
| 1. Describe the cognitive processes in diagnostic reasoning | 5 | 4 | 3 | 2 | 1 |
| 2. Define the five critical domains of diagnostic reasoning and their related components | 5 | 4 | 3 | 2 | 1 |
| 3. Apply a novel instrument to the assessment and feedback of diagnostic reasoning in a medical learner | 5 | 4 | 3 | 2 | 1 |
| ABOUT THIS ACTIVITY  Please indicate the overall effectiveness of this activity by indicating your level of agreement with the following. Please circle ONE number for EACH item.  This activity provided information that… | Strongly Agree | **Agree** | Undecided | Disagree | Strongly disagree |
| I will use in my educational activities | 5 | 4 | 3 | 2 | 1 |
| Met my personal expectations | 5 | 4 | 3 | 2 | 1 |
| Updated my current knowledge | 5 | 4 | 3 | 2 | 1 |

**Application in Practice** – circle your response

Do you intend to make practice changes? Yes	No	If so, what changes? ________________________

**TO ASSIST FUTURE WORKSHOP PLANNING, PLEASE COMPLETE THE FOLLOWING:**

1. What was most helpful to you in this teaching session?
2. What additions or changes would you make?
